# Evaluation of Task-Related Brain Activity: Is There a Role for ^18^F FDG-PET Imaging?

**DOI:** 10.1155/2019/4762404

**Published:** 2019-07-02

**Authors:** Agostino Chiaravalloti, Alessandro Micarelli, Maria Ricci, Marco Pagani, Gabriele Ciccariello, Ernesto Bruno, Marco Alessandrini, Orazio Schillaci

**Affiliations:** ^1^Department of Biomedicine and Prevention, Faculty of Medicine and Surgery, Tor Vergata University, Rome, Italy; ^2^IRCCS Neuromed, UOC Medicina Nucleare, Pozzilli (IS), Italy; ^3^Department of Clinical Sciences and Translational Medicine, Faculty of Medicine and Surgery, Tor Vergata University, Rome, Italy; ^4^Department of Radiological, Oncological and Pathological Sciences, Faculty of Medicine and Surgery, La Sapienza University, Rome, Italy; ^5^Institute of Cognitive Sciences and Technologies, CNR, Rome, Italy

## Abstract

Positron emission tomography (PET) with 2-[18F]-fluorodeoxyglucose (FDG) has been widely used for the evaluation of cortical glucose metabolism in several neurodegenerative disorders while its potential role in the evaluation of cortical and subcortical activity during a task in the healthy and pathological brain still remains to be a matter of debate. Few studies have been carried out in order to investigate the potential role of this radiotracer for the evaluation of brain glucose consumption during dynamic brain activation. The aim of this review is to provide a general overview of the applications of FDG-PET in the evaluation of cortical activation at rest and during tasks, describing first the physiological basis of FDG distribution in brain and its kinetic in vivo. An overview of the imaging protocols and image interpretation will be provided as well. As a last aspect, the results of the main studies in this field will be summarized and the results of PET findings performed in healthy subjects and patients suffering from various diseases will be reported.

## 1. Introduction

Glucose is the principal source of energy for the brain but, to date, the dynamic response of glucose utilization to changes in brain activity is still not fully understood. The brain represents only 2% of the body weight but it receives 15% of the cardiac output, 20% of total body oxygen consumption, and 25% of total body glucose utilization [1]. It was long assumed that changes in cerebral blood flow (CBF) and in the cerebral metabolic rate of oxygen (CMRO2) are tightly coupled in both resting and active brain states [2]. To date, the “neurovascular coupling” (where vasoactive metabolic products such as lactate, CO2/H+, or adenosine are responsible for increased blood flow following an increase in glucose consumption) has recently been replaced by “neuronal' hypothesis” [2]. Neuronal energy demand is communicated to the vasculature in an anticipatory, feed-forward manner by vasoactive neurotransmitters or products of synaptic signaling. Hence vasodilation occurs independently of glucose metabolism-induced signaling [2].

The brain needs an abundant and constant supply of oxygen. At rest, 80–92% fits adenosine triphosphate hails from oxidative metabolism of glucose; therefore, a functional activation, which implies the need for additional ATP and oxygen, should cause a coupled increase in both CBF and CMRO2 [3, 4]. However, previous studies about oxidative metabolism, performed with magnetic resonance imaging (MRI) or positron emission tomography (PET) imaging with ^15^O-labeled radiotracers, reported that large stimulus-induced increases in CBF were accompanied by a little increase of CMRO2. These data indicate that, during short-term functional activation, CBF and CMRO2 are not directly coupled [3, 4].

Positron emission tomography (PET) with [^18^F]-fluorodeoxyglucose (FDG) allows quantitative measurement of cerebral metabolic rates of glucose (CMRGlu). Due to its high spatial and temporal resolution, its wide distribution, and radioprotection advantages as compared to PET imaging, MRI remains in our opinion the imaging modality of choice for functional activation studies. Nevertheless, the aim of this review is to explore the current knowledge concerning PET-FDG imaging in task activation studies due to the capability of PET to explore in different biochemical aspects the possibility of performing this examination in subjects with a contraindication to MRI.

The brain tissue can be considered as a three-compartment model. The deoxyglucose-6-phosphate (DG-6-P), once formed, is essentially trapped in the tissue for the duration of the experimental procedure thus allowing obtaining images of FDG kinetics in brain 30 minutes after the injection [5–7]. In particular, considering the trend “en plateau” of the kinetic of 18F-FDG, 40% of the radiolabelled compound is extracted in the brain (~250 nCi/g) in the first minutes after the injection allowing the detection of the cortical brain areas that are activated in the first timings of the task [5–7]. Hence, FDG-PET has been considered as a useful diagnostic tool especially in the population that presented contraindication to MRI (as subjects with metallic devices (clips, vascular stents, cardiac devices, and cochlear implant), foreign bodies, tattoos containing particles of metals, or claustrophobia [8]).

Despite previous papers on physiological magnitude response to stimuli during hypoglycemia suggest otherwise [9], most of the authors assumed that glucose utilization may change in parallel with CBF and during neuronal activation. However, it is well known that in humans physiological stimulation results in a dramatic increase in cerebral blood flow and CMRGlu, especially in a short-term stimulation [10].

Instead of this dramatic increase of CMRGlu in the short-term stimulation, previous works focused on the effects of continuing visual stimulation described as well as rapid increase of CMRGlu. This raise of CMRGlu was followed by attenuation of this trend after 20 minutes of continuous stimulation that may reflect a shift from glycolytic to oxidative glucose metabolism with continued activation [11].

In order to quantify the uptake and metabolism of the tracer in tissue, a kinetic model of FDG in PET imaging, whose efficiency is demonstrated in physiological conditions in the brain, is crucial for a correct methodological approach to activation studies with PET. DG-6-P, once formed, is trapped in the tissue for a reasonable time allowing to obtain images of the brain after the injection and therefore FDG-PET can be considered as a crucial tool in evaluation of task-related brain activity [5–7].

## 2. Scanning Procedure

The measurement of CMRGlu represents neuronal processes following an intravenous injection of FDG [1]. The most of activation PET imaging studies aimed to assess changes of CMRGlu during tasks required a separate imaging session for both rest and task activation conditions. The execution of two scans lead to additional radiation with important implications in terms of radioprotection. Hence, a novel approach that required a single scan acquisition appears highly convenient. The CMRGlu, according to this procedure, may be measured at baseline and after stimuli during the same session, improving the evaluation of task-specific metabolism. Considering the intrasubject variability between repeated scan, caused by several events (e.g., caffeine, sleep status, blood chemistry changes, and misregistration), a single scan procedure may increase both sensitivity and specificity of activation PET imaging [1]. Obviously, this approach presents further undeniable advantages in terms of costs, radioprotection, and patient's compliance (assuming that the specific task may be performed in the nuclear medicine unit).

The widely used clinically method, used both in single and in two scans PET imaging studies, is characterized by a scan performed 20-40 minutes after a bolus FDG injection. The bolus method may provide a quantitative and/or qualitative measurement of the CMRGlu after stimuli. Indeed, in most of preceding activation, PET studies used a bolus injection of FDG in order to characterize functional cortical metabolic responses to multiple and different kinds of stimulation, including visual, auditory, cognitive tasks, and drug administration. This bolus method reports an integral of neuronal processes during 20-40 minutes. However, considering the trend “en plateau” of the kinetic of FDG and that the 40% of the radio-labeled compound is extracted in the brain (~250 nCi/g) in the first minutes after the injection, the CMRGlu after bolus injection may be a mirror, in particular of initial metabolic activation [5, 6]. Hence, some limitations regarding the lack of information about dynamic metabolic changes during the scan and/or during the task tend to affect this method which allows the detection especially of the cortical brain areas that are activated in the first timings of our task.

Recently, a novel technique with a constant infusion of FDG for the entire scan was proposed [1, 12]. Advantages of constant radioligand infusion are to avoid the prominent FDG uptake in the early period and to provide available radioligand during the entire execution of the task. These kinetic aspects may improve the quantification of the dynamic metabolic changes in the brain during the task, promoting a single scan procedure that allows measuring in the same session CMRGlu at baseline and during the task execution [1, 12].

In order to minimize interindividual differences, a Statistical Parameter Mapping (SPM) analysis (see below) may be generally useful in dynamic PET imaging considering physiological variations of CMRGlu at baseline and, even more so, a previous paper evaluated effects of age and sex during dynamic PET study. Authors in fact compared rest glucose metabolism to verbal memory task activation in healthy subjects and described both age-related metabolic decline and sex differences within frontal regions that resulted more markedly in medial frontal and cingulate areas. This pattern is consistent with some age-related patterns of affective and cognitive change [13].

In activation PET imaging studies a quantitative analysis should be preferred over a qualitative visual analysis of images. An accurate quantification of PET measurements can be obtained by measurement of tracer concentration through blood sampling. This procedure may result in being uncomfortable for the patient and therefore can partially be avoided with the comparison of FDG uptake in case studies to that of a group of subjects that serve as control group.

## 3. Task-Related Procedure

To date, FDG-PET imaging was used for the evaluation of CMRGlu during several and different types of tasks, in order to evaluate a physiological and/or pathological response to stimuli. A general overview is provided in Table 1. PET imaging may thus improve the diagnostic and therapeutic approach and/or evaluate the efficiency of treatment. Potentially activation PET imaging with FDG may be useful in both physiological studies of the brain and in most of the diseases, affecting glucose metabolism and CBF but the procedure should be conformed to specific experiment. The experimental procedure may vary based on the population enrolled (i.e., healthy subjects, patients affected by various types of brain damage, or those affected by neurodegenerative diseases) and the types of tasks (visual, auditory, somatic, etc.).

In the example, motor task studies in controls subjects required cortical activity at rest and during motor activation to be assessed with FDG-PET on two consecutive days [14], while in a force and position task study, each participant was injected with FDG and PET imaging was performed immediately after both tasks [15]. Moreover, in human volitional swallowing task, PET scans were obtained in all subjects on two separate occasions: subjects were initially randomized to one of two conditions: swallowing or rest. The alternate condition was performed for the follow-up scan on a separate day, at least seven days later [16]. Task-related procedures as word repetition and word association or during lexical decision or after learning a complex visuospatial/motor task were evaluated with FDG-PET as well [17–19]. Interestingly, as compared with word repetition, word association was associated with significant increases in CMRGlu in the left prefrontal cortex, the left frontal operculum (Broca's area), and the left insula, indicating the involvement of these areas in associative language processing. Decreased CMRGlu was found in the left posterior cingulum during word association. During word repetition, highly significant negative correlations were found between the left prefrontal cortex, the contralateral cortex areas, and the ipsilateral posterior cingulum [18].

In a recently published paper, Zwergal A et al. investigated the spatial orientation during a horizontal and vertical real navigation task in humans [20]. In this paper, FDG was injected at the start of the 10 min spatial orientation paradigm and image acquisition started 30 min after tracer administration [20]. Contrarily, in functional brain mapping of actual car driving, the injection and the imaging have been performed after task [21]. Therefore considering the differences between tasks the procedures, particularly the duration and timing of the task in relation to injection, should conform to specific experiment.

Nevertheless, a previous paper that investigated the variations of cortical CMRGlu in olfactory processing proposed in this case a methodological approach. In this specific activation-task procedure, the relatively ecological olfactory environment at rest could represent a limitation. Therefore, authors considered rest as neutral olfactory stimulation in order to avoid olfactory pollution at rest and, after a month, compared to pure olfactory task activation scan. A comparison of FDG uptake after a bolus injection at rest and during stimulation has been performed [22].

## 4. Analysis of Pet Data

SPM (https://www.fil.ion.ucl.ac.uk/spm/) is a tool widely used for analysis of PET data in most of the recent imaging studies considered in this review [14, 15, 23–27]. In these studies, this tool has been used for comparison of a group of patients (i.e., before and after tasks), but recently has been also proposed for the testing of a single subject by comparison with a reference group [28].

PET data are usually converted from DICOM to Nifti format using Mricron software available at https://www.nitrc.org/projects/mricron and then subjected to a normalization process. The normalization includes several steps as bias regularization; affine registration with the tissue probability maps [29] is used to achieve approximate alignment to ICBM space template: European brains [30, 31], warping, smoothing, and application of Gaussian filter. Recently Della Rosa et al. developed a new FDG-PET aging and dementia-specific template for spatial normalization, based on images derived from both age-matched controls and patients [32]. This template increases the spatial normalization accuracy of PET data and can improve the SPM analyses performed on patients with neurodegenerative diseases [32]. Before analyses, usually a global normalization and a transformation tool of statistical parametric maps into a normal distribution and correction of SPM coordinate to match the Talairach coordinates; subroutine implemented by Matthew Brett (http://www.mrc-cbu.cam.ac.uk/Imaging) is applied. Brodmann areas (BA) are usually identified at a range from 0 to 3 mm from the corrected Talairach coordinates of the SPM output isocenter by using a Talairach client available at http://www.talairach.org/index.html. As proposed by Bennett et al. [33], SPM t-maps are usually corrected for multiple comparisons with the false discovery rate (P≤0.05) and corrected for multiple comparisons at the cluster level (P≤0.001)[22–25].

Signorini et al. highlighted the usefulness of SPM in the objective assessment (including localization in stereotactic space) of regional glucose consumption abnormalities in patients with degenerative or developmental disorders, including probable Alzheimer's disease, progressive aphasia, multiple sclerosis, developmental specific language impairment, and epilepsy [34]. SPM analysis showed higher sensitivity and specificity (96% and 84%) and better diagnostic positive and negative likelihood ratios as compared to clinical evaluation and qualitative evaluation of FDG-PET Images in the detection of dementia. Moreover, SPM analysis increased diagnostic accuracy for differential diagnosis of dementia [28].

## 5. Results in Healthy Controls

Several PET imaging studies were performed in healthy subjects during tasks in order to evaluate brain metabolic response to several kinds of stimuli in physiological conditions.

Activation PET imaging was performed in order to evaluate task-related changes in the visual cortex. An increased glucose metabolic rate was described during a visual task in the human primary and associative visual cortex, as the complexity of visual scenes increased [35].

PET imaging with FDG was performed also to describe the cortical metabolic response to motor stimuli. In these cases, during a motor task imaging reported significantly increased motor-associated activation of the contralateral primary motor cortex, the supplementary motor area, and the ipsilateral (right) cerebellum [14]. Furthermore, a similar paper that focused on the comparison of different motor tasks (force and position motor tasks) described different FDG uptake increase patterns depending on stimuli required [15], validating the clinical role of these tasks to define the function of specific brain areas.

Furthermore, PET imaging was performed during volitional swallowing, with possible implications in the evaluation of risks related to swallowing affection after brain injury and in personalized rehabilitative strategies [16].

Future application of these findings in motor tasks may lead to ameliorating diagnosis protocols of those diseases associated with motor cortex disorders. Moreover, activation PET imaging may improve designs of therapeutic and rehabilitative strategies after several brain pathologies.

For instance, CMRGlu was measured in eight healthy subjects performing a complex visuospatial/motor task (the computer game Tetris), before and after weeks of practice. Subjects who most improved their Tetris performance after practice showed the largest glucose metabolic decreases after practice in several areas. This could suggest that learning may result in decreased use of extraneous or inefficient brain areas that may reflect changes in cognitive strategy that are a part of the learning process [17]. Of interest, PET procedures have been combined in order to depict those functions addressed to neuroanatomical grounds related to the nose and its related roles. A comparison of FDG uptake after a bolus injection at rest and during stimulation was performed in 11 normosmic subjects and reported, at rest, mainly increased glucose metabolism in left superior, inferior, middle, medial frontal, orbital gyri, and anterior cingulate cortex. Conversely, during olfactory stimulation PET imaging reported hyperactivation in the cuneus, lingual, and parahippocampal gyri, mainly in the left hemisphere [22, 23].

CMRGlu was evaluated in healthy subjects also in more complex paradigms such as passive problem solving [36], the visual lexical decision [19], and car driving [21], describing, respectively, activated cortical areas. Other brain functions were tested by PET imaging as spatial orientation function (tested during a horizontal and vertical navigation task), indicating a functional anisotropy of human-3D navigation in favor of the horizontal plane [20].

Furthermore, activation PET imaging was performed in healthy subjects during word repetition when compared to word cognitive association in order to evaluate hyperactivated cortical areas associated with an intracerebral functional network of language and cognitive process [18]. Activation PET imaging with FDG was performed to evaluate major emotion-recognition related brain areas response to emotional stimuli. In this case, the increase of glucose metabolism has been reported during facial emotion-recognition task in human amygdala. In particular, activation of the left amygdala was described during a continuous emotional task in 10 healthy subjects. Furthermore, in 8 healthy volunteers, increased metabolism of right amygdala was described watching emotional videos [37, 38].

These studies were carried out on a few patients and more trials on a large population are necessary; nevertheless, the results in emotion-recognition function are promising and may be certainly useful in the psychiatric field.

In particular, the role of the amygdala during facial emotion-recognition tasks as well as its clinical implications in schizophrenia patients remains unclear. These findings allow a better knowledge of physiological cortical metabolic response to stimuli and they may eventually improve the diagnostic and therapeutic approach to brain physiopathology [38].

## 6. Results in Patient Population

Previous studies investigated the variations of cortical CMRGlu in olfactory processing in 26 patients affected by multiple chemical sensitivity (MCS) that usually react to odor compounds and, compared to normosmic findings, describing a different activation metabolic pattern of FDG uptake in MCS [24].

During olfactory stimulation, an increase of glucose metabolism at PET imaging was described in bilateral amygdala, olfactory cortex, caudate, and pallidum associated with a decrease of metabolism in bilateral putamen and hippocampus both in MCS and in controls. Nevertheless, in MCS a significant higher metabolism in the bilateral olfactory cortex was described also at rest, defining a particular pathological pattern of CMRGlu and depicting for the first time a subcortical resting state correlates of hypersensitized neural structure [24].

Furthermore, FDG uptake during stimulation was compared to odor pleasantness scale in both groups. In normosmic individuals, CMRGlu showed negative correlations in bilateral amygdala and hippocampus while in MCS subjects CMRGlu resulted in being positively correlated to odor pleasantness scale in bilateral putamen, supporting the hypothesis that in MCS the same olfactory-related function could be shifted and hosted in diverse regions with respect to controls[25].

FDG-PET imaging during a visual language task was performed in 6 prelingually deafened children in order to provide objective information on the development and plasticity of cortical language networks. The widest activated cortical area was described in the worst user of spoken language while, in the best user of spoken language, CMRGlu resulted in being not significantly different to healthy controls [39], whereas further studies are needed these findings depict a promising role of activation PET imaging in rehabilitation and education of prelingually deafened children, allowing a possible improvement of strategies in terms of therapy and communication [39].

Dynamic PET studies were performed also in patients affected by Alzheimer's disease. A single and specific alteration in FDG metabolism has not been identified in this form of dementia and, therefore, the FDG-PET hypometabolic pattern is assumed to be the result of some combination of processes involved in its own pathogenesis [40]. The anatomy of the AD signature includes precuneus and posterior cingulate gyri, the inferior parietal lobule, posterolateral portions of the temporal lobe, and the hippocampus and medial temporal cortices. Metabolic reduction usually gets worse throughout the course of the disease, often describing bilateral asymmetry at early stages [40].

Activation PET imaging with FDG was performed in Alzheimer's disease patients during different types of the task, describing the response of a pathological brain to several different stimuli. In a previous study, a continuous visual recognition task was compared to rest in Alzheimer disease patients resulting in such findings that support the hypothesis for which metabolic rate at rest reflects the extent of morphological damage. On the other side, PET studies during activation indicate the brain's reserve capacity to respond to functional tasks, suggesting that activation PET imaging could help to select patients that may be more responsive to therapy [41].

PET imaging studies during activation with FDG in AD patients were also used to describe specific cortical metabolic patterns during an episodic learning and recognition tasks, reflecting auto noetic consciousness. Activation PET imaging aims were to describe hyperactive specific cortical networks associated with task activation and to validate, as well, the clinical role of the common neuropsychological test battery. The found correlations indicated that the impairment of episodic memory is mainly subserved by the dysfunction of frontal areas and of the hippocampal region [42].

In the psychiatric field, dynamic PET imaging was performed to assess CMRGlu in several diseases. One emerging hypothesis regarding psychiatric illnesses is that they arise from the dysregulation of normal circuits or neuroanatomical patterns that may be associated with an abnormal CMRGlu pattern. Activation PET studies were performed during an auditory continuous performance task in unipolar, bipolar, and healthy individuals. In healthy subjects a general pattern with the metabolism of cortical regions being inversely related to that of subcortical structures, particularly the frontal cortex with the cerebellum, amygdala, and thalamus, was described. Conversely, an abnormal pattern of FDG uptake was reported in the bipolar and unipolar disease when compared to healthy controls and it suggested marked interregional neuronal dysregulation in bipolar and unipolar illness [43, 44]. These findings may allow the improvement of diagnostic and therapeutic approach providing a rationale for using acute and long-term therapies.

In activation PET studies, left amygdala hyperactivation among the schizophrenia group was described in both emotional and control tasks when compared to controls, instead of the right amygdala that showed no differential activation in any of the tasks. Patients diagnosed with schizophrenia exhibit a non-task-specific amygdala hyperactivation during a continuous emotional and nonemotional task [45].

PET imaging with FDG seems particularly promising during the early course of schizophrenia, in order to improve the diagnostic and therapeutic approach. The performance of acute first episode schizophrenia cases on the correlation of a facial emotion perception task was evaluated with PET imaging. Schizophrenia subjects showed hypometabolism in brain regions implicated in emotion processing compared to controls and a positive correlation of prefrontal CMRGlu and performance indices on emotions domain was demonstrated [27].

## 7. Results in Drug Testing

Activation PET imaging was also performed during drug administration tasks. Regular marijuana smokers were evaluated during virtual reality maze task with and without administration of THC. Imaging with THC showed increased CMRGlu in areas associated with motor coordination and attention. On the other side, reduced CMRGlu in areas related to visual integration of motion was found when compared to results without THC, possibly suggesting an impact on cognitive-motor networks [46]. PET imaging was also performed in chronic methamphetamine abusers in the first days and after a few weeks of abstinence, during the performance of a vigilance task. Global glucose metabolism increased in methamphetamine-dependent, particularly in neocortex reporting a shift in cortical-subcortical metabolic balance, with a maximal increase (>20%) in parietal regions. Parietal CMRGlu in methamphetamine-dependent resulted in being related to changes in reaction time and self-reports of negative affect whereas ventral striatum CMRGlu resulted to be related to changes in self-reports of depressive symptoms demonstrating great relevance in treatment success evaluation due to the role of this region in drug abuse-related behaviors [26].

These findings suggest the relevance of activation PET imaging with FDG in drugs-related changes in specific networks, evaluating the effect on brain metabolism during a specific task. Thus, PET information may influence a personalized therapeutic approach, based also on neuroimaging variations during therapy.

## 8. Task-Related Functional Magnetic Resonance Imaging (fMRI)

Functional magnetic resonance imaging (fMRI) is a diagnostic technique that is based on blood-oxygen-level-dependent (BOLD) contrast changes (usually induced by task-related activities) or in the measure of the subjects' baseline BOLD variance [47, 48]. The first that allows the detection of regional, time-varying changes in brain metabolism is mostly associated with local changes in blood flow [47, 49]; as reported in the introduction section, the use of a cortical area is related to a local increase of blood flow [50]. Resting State fMRI uses those signals that are discarded in task fMRI studies and that represents spontaneous fluctuations and are confined to distinct cortical network systems in the brain; in other terms, it analyses the spontaneous BOLD signal in the absence of any explicit task or an input [48].

As compared to other imaging modalities used for activation studies as PET or electroencephalography (EEG), the advantages of fMRI are represented by a high spatial resolution. Moreover, scanners for fMRI are widely distributed radiology facilities. The fMRI pixel size is 3–4 mm that is significantly small as compared to PET (ranging from 5 to 10 mm) or EEG (20 mm) [51]. As for temporal resolution, typically the BOLD response has a width of ~3s and a peak occurring ~5–6s after the onset of a brief neural stimulus [47]. Together with the limitations mentioned previously on biodistribution of PET radiotracers in human tissues, a PET scan for the brain may require more than ten minutes to be completed and, most important, changes in neural processes can only be studied by repeating the scan. For these reasons, fMRI remains in our opinion the imaging modality of choice for brain imaging in functional activation studies.

## 9. Conclusions

Evaluation of task-related brain activity by means of FDG-PET is a promising diagnostic tool for the evaluation of cortical and subcortical activity in healthy subjects and patients with various diseases during tasks. The sensitivity of this imaging modality is enhanced by the use of novel techniques as SPM in the analysis of PET data. Nevertheless, this diagnostic technique has several limitations: (a) the relative slow kinetic of FDG in brain that may limit the detection of cortical and subcortical areas that are rapidly activated during stimuli and may increase the detection of those areas that are lately activated/deactivated during stimuli and prior to images acquisition; (b) ionizing radiation exposure that should be considered carefully especially in young individuals.

## Figures and Tables

**Table 1:** Summary of the most relevant studies on PET imaging with FDG in different types of tasks.

Task	Number of subjects	Type of PET imaging modality	Outcome	Reference
Visual task	5 subjects	Visual stimulation was started 5 minutes before injection of FDG and continued throughout the study.	Hypermetabolism in the primary and associative visual cortex, as the complexity of visual scenes increased.	[35]

Motor task	1 subject	The volunteer underwent 20 min periods of rest and motor activation (motor task, which involved repetitively grasping and releasing the right hand, performed during the initial 5 min of the activation period)	Hypermetabolism in the contralateral primary motor cortex, the supplementary motor area, and the ipsilateral (right) cerebellum.	[14]

Force and position motor tasks	2 subjects	FDG injection has been performed during both a force and position task (separated by 7 days, with the elbow flexor muscles at 15% maximal voluntary contraction force)	Greater metabolism in the occipital and temporal cortices of the brain during the position task compared to the force task.	[15]

Volitional swallowing task	8 male subjects	PET-FDG at rest and while swallowing (20-second intervals for 30 minutes) in the erect seated position	During swallowing hypermetabolism in left sensorimotor cortex, cerebellum, thalamus, precuneus, anterior insula, left and right lateral postcentral gyrus, and left and right occipital cortex; decreased metabolism in the right premotor cortex, right and left sensory and motor association cortices, left posterior insula and left cerebellum.	[16]

Complex visuospatial/motor task	8 male subjects	Subjects underwent PET-FDG performing a complex visuospatial/motor task (the computer game Tetris), before and after 4–8 weeks of daily practice on Tetris	After practicing glucose metabolism in cortical surface regions decreased despite an increased performance: subjects who improved their Tetris performance the most after practice showed the largest glucose metabolic decreases in several areas. These results suggest that learning may result in decreased use of extraneous or inefficient brain areas.	[17]

Osmic task	11 subjects; 26 subjects	Injection of FDG in bolus during neutral (rest) and during a pure (stimulation) olfactory condition in both papers.	At rest mainly increased glucose metabolism in left superior, inferior, middle, medial frontal, orbital gyri and anterior cingulate cortex, while during olfactory stimulation hyperactivation in the cuneus, lingual, and parahippocampal gyri, mainly in the left hemisphere in both papers.	[22, 23]

Passive problem solving tasks	22 subjects	PET-FDG have been performed while the subjects viewed videos on two occasions, tasks with no inherent reasoning or problem solving, and data have been compared to Raven's Advanced Progressive Matrices test (RAPM) scores.	Hyperactivation in specific posterior brain areas in high RAPM scorers. Subsequent analyses revealed a high/low RAPM group difference in functional connectivity between left activity and the left anterior cingulate/medial frontal gyrus. These data provide evidence that individual differences in intelligence correlate to brain function even when the brain is engaged in nonreasoning tasks.	R. J. Haier, N. S. White and M. T. Alkire, “Individual differences in general intelligence correlate with brain function during nonreasoning tasks,” *Intelligence*, vol. 31, no. 5, pp. 429-441, 2003.

Visual lexical decision tasks	11 right-handed male subjects	Subjects underwent PET-FGD during rest and two versions of a visual lexical decision task ( novel or repeated stimuli performed during the 30 min FDG uptake period).	Comparisons of the tasks with the resting condition revealed significant relative activations of visual and motor areas. Novel words evoked significantly greater activation in the left and right anterior cingulate gyri and right hippocampal formation than did repeated words in the same task. Relative glucose metabolism in the left angular gyrus was significantly greater to novel words than to resting. Thus, two tasks equated in sensory, motor, and decision processes, but differing in the familiarity of the stimuli, evoke significantly different patterns of brain activation.	V. I. Nenov, E. Halgren, M. Mandelkern and M. E. Smith, “Human brain metabolic responses to familiarity during lexical decision,” *Hum Brain Mapp*, vol. 1, no. 4, pp. 249-268, 1994.

Car driving task (active and passive driving)	30 subjects	Subjects have been divided into three subgroups for examination by PET-FDG: active driving (10 subjects that drove for 30 minutes), passive driving (10 subjects that participated as passengers on the front seat) and control (10 subjects remained seated in a lit room) groups.	In active driving: hyperactivation in the primary and secondary visual cortices, primary sensorimotor areas, premotor area, parietal association area, cingulate gyrus, the parahippocampal gyrus, thalamus and cerebellum. In passive driving: manifested a similar-looking activation pattern, lacking activations in the premotor area, cingulate and parahippocampal gyri and thalamus. Direct comparison of the active and passive driving conditions revealed activation in the cerebellum.	M. Jeong, M. Tashiro, L. N. Singh, K. Yamaguchi, E. Horikawa, M. Miyake, S. Watanuki, R. Iwata, H. Fukuda, Y. Takahashi and M. Itoh, “Functional brain mapping of actual car-driving using [18F]FDG-PET,” *Ann Nucl Med*, vol. 20, no. 9, pp. 623-628, 2006.

Horizontal and vertical navigation tasks in real space	24 right-handed male subjects	Spatial orientation was tested during a horizontal and vertical real navigation task. Video tracking of eye movements was used to analyse the behavioral strategy and combined with simultaneous measurements of brain activation and metabolism.	During horizontal navigation metabolism increased in the right hippocampus, bilateral retrosplenial cortex, and pontine tegmentum; during vertical navigation in bilateral hippocampus and insula. Direct comparison revealed a relative activation in the pontine tegmentum and visual cortical areas during horizontal navigation and in the flocculus, insula, and anterior cingulate cortex during vertical navigation.	A. Zwergal, F. Schoberl, G. Xiong, C. Pradhan, A. Covic, P. Werner, C. Trapp, P. Bartenstein, C. la Fougere, K. Jahn, M. Dieterich and T. Brandt, “Anisotropy of Human Horizontal and Vertical Navigation in Real Space: Behavioral and PET Correlates,” *Cereb Cortex*, vol. 26, no. 11, pp. 4392-4404, 2016.

Word repetition and word cognitive association tasks	8 subjects	Two different tasks were performed in randomized order during PET FDG scanning: word repetition (after the auditory presentation of nouns) as a control condition, and word association (after the auditory presentation of nouns) as a specific semantic activation.	Word association was associated with Activation in the left prefrontal cortex, the left frontal operculum (Broca's area) and the left insula, indicating the involvement of these areas in associative language processing. decreased metabolism was found in the left posterior cingulum during word association; during word repetition, highly significant negative correlations were found between the left prefrontal cortex, the contralateral cortex areas, and the ipsilateral posterior cingulum.	M. Schreckenberger, E. Gouzoulis-Mayfrank, O. Sabri, C. Arning, G. Schulz, T. Tuttass, G. Wagenknecht, H. J. Kaiser, H. Sass, and U. Buell, “Cerebral interregional correlations of associative language processing: a positron emission tomography activation study using fluorine-18 fluorodeoxyglucose,” Eur J Nucl Med, vol. 25, no. 11, pp. 1511-1519, 1998.

Continuous emotion task	10 subjects	PET FDG performed during facial emotion recognition studies and neutral on a different day	Activation the left amygdala and activation of the emotional recognition-related areas	E. Fernandez-Egea, E. Parellada, F. Lomeña, C. Falcon, J. Pavia, A. Mane, G. Sugranyes, M. Valdes, M. Bernardo, “A continuous emotional task activates the left amygdala in healthy volunteers: (18)FDG-PET study,”Psychiatry Res. 2009 Mar 31;171(3):199-206

Long-term, free recall of emotional information	8 right-handed male subjects	Subjects viewed two videos during PET scanning, separated by 3-7 days, consisting either of emotionally arousing film clips or of neutral film clips. Three weeks after the second session, memory for the videos was assessed in a free recall test.	Glucose metabolic rate of the right amygdaloid complex while viewing the emotional films was highly correlated with the number of emotional films recalled and was not correlated with the number of neutral films recalled.	L. Cahill, R. J. Haier, J. Fallon, M. T. Alkire, C. Tang, D. Keator, J. Wu, and J. L. McGaugh, “Amygdala activity at encoding correlated with long-term, free recall of emotional information,” Proceedings of the National Academy of Sciences of the United States of America, vol. 93, no. 15, pp. 8016-8021, 1996.

## Abbreviations

CBF: Cerebral blood flow
CMRO2: The cerebral metabolic rate of oxygen
CMRGlu: Cerebral metabolic rates of glucose
FDG: 2-[18F]-fluorodeoxyglucose
DG: Deoxyglucose
DG-6-P: Deoxyglucose-6-phosphate
SPM: Statistical parametric mapping.
